# Integrated transcriptome sequencing and dynamic analysis reveal carbon source partitioning between terpenoid and oil accumulation in developing *Lindera glauca* fruits

**DOI:** 10.1038/srep15017

**Published:** 2015-10-08

**Authors:** Jun Niu, Yinlei Chen, Jiyong An, Xinyu Hou, Jian Cai, Jia Wang, Zhixiang Zhang, Shanzhi Lin

**Affiliations:** 1 College of Biological Sciences and Biotechnology, College of Nature Conservation, National Engineering Laboratory for Tree Breeding, Key Laboratory of Genetics and Breeding in Forest Trees and Ornamental Plants, Ministry of Education, Beijing Forestry University, Beijing 10083, China

## Abstract

*Lindera glauca* fruits (LGF) with the abundance of terpenoid and oil has emerged as a novel specific material for industrial and medicinal application in China, but the complex regulatory mechanisms of carbon source partitioning into terpenoid biosynthetic pathway (TBP) and oil biosynthetic pathway (OBP) in developing LGF is still unknown. Here we perform the analysis of contents and compositions of terpenoid and oil from 7 stages of developing LGF to characterize a dramatic difference in temporal accumulative patterns. The resulting 3 crucial samples at 50, 125 and 150 days after flowering (DAF) were selected for comparative deep transcriptome analysis. By Illumina sequencing, the obtained approximately 81 million reads are assembled into 69,160 unigenes, among which 174, 71, 81 and 155 unigenes are implicated in glycolysis, pentose phosphate pathway (PPP), TBP and OBP, respectively. Integrated differential expression profiling and qRT-PCR, we specifically characterize the key enzymes and transcription factors (TFs) involved in regulating carbon allocation ratios for terpenoid or oil accumulation in developing LGF. These results contribute to our understanding of the regulatory mechanisms of carbon source partitioning between terpenoid and oil in developing LGF, and to the improvement of resource utilization and molecular breeding for *L. glauca*.

*Lindera glauca*, a member of the family Lauraceae and the genus *Lindera Thunb*, is widely distributed in the forests at low altitudes only in China, Japan and Korea^1^,^2^,^3^,^4^,^5^. In China, this plant is one of the most economically and ecologically important and intensively studied tree species owing to its very plentiful resource, superior adaptability, ecological benefits and especially abundant terpenoid contents^2^,^3^,^4^,^5^,^6^,^7^,^8^,^9^. More recently, the abundance of oil (approximately 15% of fresh weight) has been characterized mainly with capric acid (11.83%), palmitic acid (30.13%), oleic acid (36.18%) and linoleic acid (12.92) in LGF^1^. Thus, LGF has recently emerged as a novel specific experimental material to explore the regulatory mechanism of high-quantity accumulation of terpenoid and oil in plants. Importantly, the precursors required for either terpenoid or oil biosynthesis are mostly from glycolysis and pentose phosphate pathway (PPP)^10^,^11^, thus the complex and interactional regulatory mechanisms of carbon partitioning between terpenoid and oil in developing LGF.

Carbon is supplied to the heterotrophic organs (fruits, seeds, roots and tubers) mostly as sucrose from photosynthetic tissues, and sucrose synthase (SuSy) or sucrase (SUC) catalyze the cleavage of sucrose to hexoses (glucose and fructose) in cell wall^12^, which are then converted to glyceraldehyde 3-phosphate (G3P) via PPP or pyruvate (PYR) via the glycolysis in both cytosol and plastid, as carbon source for terpenoid and oil biosynthesis^10^,^11^. In general, the interchange of glycolytic intermediates between cytosol and plastid in plants has been implicated in the highly selective transporters, including glycolipid transporter (GLT), glucose-6-phosphate transporter (GPT), triose phosphate transporter (TPT) and phosphoenolpyruvate transporter (PPT)^13^,^14^,^15^. Additionally, because biological membranes are impermeable to acetyl-CoA as an important precursor for *de novo* fatty acid (FA) biosynthesis in plastids, terpene biosynthesis and FA elongation in cytosol, this molecule must be synthesized within each subcellular compartment by plastidial PYR dehydrogenase complex (PDHC), mitochondrial PDHC or cytosolic ATP-citrate lyase (ACL)^16^,^17^. Thus, the multiple mechanisms of generating acetyl-CoA for different acetyl-CoA-requiring metabolisms indicate the complexity of carbon flux into TBP and OBP in developing LGF.

In higher plants, the identifications of functional enzymes involved in the biosynthesis of terpenoid (http://www.genome.jp/kegg-bin/show_pathway?map01062) and oil (http://aralip.plantbiology.msu.edu) have been well facilitated by various experimental methods^18^,^19^,^20^,^21^,^22^,^23^,^24^,^25^,^26^,^27^,^28^,^29^,^30^,^31^,^32^,^33^,^34^,^35^,^36^,^37^,^38^. There are two pathways for terpenoid backbone biosynthesis, cytosolic mevalonate (MVA) and plastidial 2-Cmethyl-Derythritol 4-phosphate (MEP) pathway, in which the 1-deoxy-D-xylulose-5-phosphate synthase (DXS) and hydroxymethylglutaryl-CoA reductase (HMGCR) are well characterized as the rate-limiting enzymes in MEP and MVA pathway, respectively^18^,^19^. By geranyl diphosphate synthase (GPPS) and farnesyl diphosphate synthase (FPPS), the produced precursors of isopentenyl diphosphate (IPP) and dimethylallyl diphosphate (DMAPP) in MEP and MVA pathway are respectively catalyzed to geranyl diphosphate (GPP) and farnesyl diphosphate (FPP), as the important precursors required for monoterpenoids (C10) biosynthesis by monoterpene synthase (mono-TPS) and sesquiterpenoids (C15) biosynthesis by sesquiterpene synthase (sesqui-TPS), respectively^20^,^21^,^22^,^23^. As for oil biosynthesis, carboxylation of acetyl-CoA to malonyl-CoA has been well known as the first rate-limiting step in FA synthesis by a multi-subunit acetyl-CoA carboxylase (ACCase) complex, including carboxyltransferase (CT), biotin carboxylase (BC) and biotin carboxyl carrier protein (BCCP)^13^,^24^,^25^,^26^. Before entering the synthesis pathway of FAs, malonyl-CoA from acetyl-CoA is firstly transferred to malonyl-ACP by malonyl-CoA: ACP malonyltransferase (MCMT) to provide two-carbon unit at each step of elongation^25^,^26^. There are four reactions of condensation, reduction, dehydration and reduction occurred in each synthesized cycle, which is catalyzed by a series of regulatory enzymes, including ketoacyl-ACP Synthase III (KASIII), ketoacyl-ACP reductase (KAR), hydroxyacyl-ACP dehydrase (HAD), enoyl-ACP reductase (EAR) and KASI^25^,^26^. After 7 elongated cycles, the produced saturated C16:0-ACP can either be hydrolyzed by fatty acyl-ACP thioesterase B (FATB) to release free FAs or elongated by KASII to generate 18:0-ACP. Subsequently, the produced 18:0-ACP is desaturated to 18:1-ACP by stearoyl-ACP desaturase (SAD), and then 18:1-ACP be hydrolyzed to free C18:1 by FATA^26^. The resulting free FAs are exported from plastid to endoplasmic reticulum (ER), and converted to fatty acyl-CoAs by long-chain acyl CoA synthetase (LACS), as the substrate for esterification and elongation^26^,^27^. In ER, fatty acyl-CoAs can be further elongated sequentially by ketoacyl-CoA synthase (KCS), ketoacyl-CoA reductase (KCR1), hydroxyacyl-CoA dehydratase (HCD) and enoyl-CoA reductase (ECR) to produce long chain FAs (>18 carbon)^26^,^28^. TAG assembly from G3P and acyl-CoAs involves four enzymatic steps: first, two acylations of G3P by glycerol-3-phosphate acyltransferase (GPAT) and lysophosphatidic acid acyltransferase (LPAAT), followed by phosphatidic acid phosphatase (PAP), and a third acylation by diacylglycerol acyltransferase (DGAT) or phospholipid:diacylglycerol acyltransferase (PDAT)^29^,^30^,^31^,^32^,^33^,^34^,^35^. During the TAG synthesis, CDP-choline:1,2-sndiacylglycerol cholinephosphotransferase (CPT) and phosphatidylcholine: diacylglycerol cholinephosphotransferase (PDCT) was characterized that exchanges acyl residues between DAG and phosphatidylcholine (PC) for further desaturated by FA desaturase 2/3(FAD2/3)^37^,^38^. All these findings have shown one highly complex process involved in a series of regulatory enzymes for terpenoid and oil biosynthesis in plants. Although considerable progress of *L. glauca* has been made in the contents and compositions of terpenoid and oil^2^,^3^,^4^,^5^,^6^,^7^,^8^,^9^, literature and molecular resources available for *L. glauca* remain very scarce.

As one of deep sequencing technology, RNA sequencing without recourse to the genomic information has been widely applied for extensive and intensive transcriptional studies in many plant species, such as *Salvia miltiorrhiza*, *Liriodendron chinense*, *Elaeis guineensis*, *Phoenix dactylifera*, *Brassica napus*, *Ricinus communis*, *Euonymus alatus* and *Tropaeolum majus*^39^,^40^,^41^,^42^. More recently, our transcriptomic analysis for different tissues of *L. glauca* has been performed by Illumina transcriptome sequencing^43^, but the obtained data are not still suitable for us to deeply explore the comprehensive regulatory mechanisms of carbon source partitioning between the terpenoid and oil accumulation in developing LGF. Thus, the transcriptomic analysis of developing LGF has become an imperative.

In the current study, we used the LGF as a novel specific experimental material, and analyzed the temporal patterns for the contents and compositions of terpenoid and oil at 7 different developing stages (25, 50, 75, 100, 125, 150 and 175 DAF). In result, the representative periods were characterized for comparative deep transcriptomic analysis by using Illumina technology. After functional annotation and classification, the genes involved specifically in glycolysis, PPP, TBP and OBP were identified in developing LGF. We then focus on combining differential expression profiling and qRT-PCR to analysis the transcriptional levels for some key enzymes and transcription factors (TFs) associated with the regulation of carbon allocation ratios, highlighting the complexity of alternative carbon flux for terpenoid and oil accumulation in developing LGF for the first time.

## Results

### Different accumulative patterns of terpenoid and oil in developing LGF

**Figure 1:**
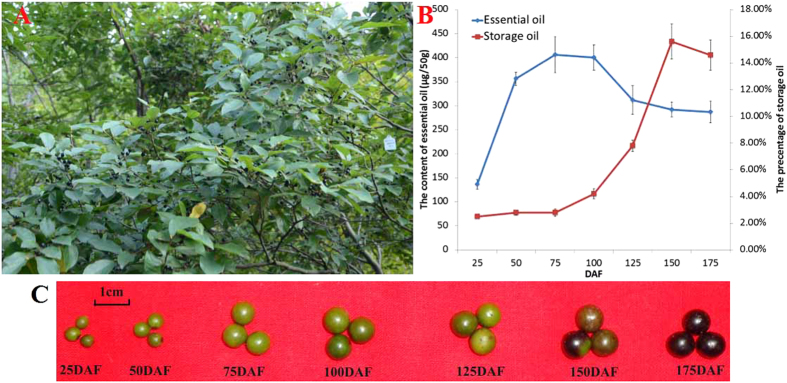
The features of *Lindera glauca* fruits. (**A**) The *Lindera glauca* are fruiting. (**B**) The dynamical patterns for LGF oil and FAs at different development period. (**C**) 7 developing stages of LGF.

We systematically measured the contents and compositions of terpenoid and oil during the whole developmental stages of LGF from 25 DAF (immature stage) to 175 DAF (fully matured stage) for the first time. The terpenoid contents showed a significant increase from 25 DAF (136.05 ± 9.99 μg/50 g) to 75 DAF (406.09 ± 36.97 μg/50 g), and then a decrease from 100 DAF (404.19 ± 26.33 μg/50 g) to 175 DAF (287.06 ± 22.03 μg/50 g), while oil contents displayed a gradual increase from 25 DAF (2.50 ± 0.13%) to 150 DAF (15.60 ± 1.30%) and then an approximately 1% decline at 175 DAF (Fig. 1B), indicating terpenoid and oil accumulation at the early and middle-late stages during developing LGF, respectively. By GC-MS for the dynamic changes of the terpenoid and oil compositions, we characterized all the terpenoids (mainly including α-pinene, β-cis-ocimene, eucalyptol, (Z)-β-farnesene and α-selinene) with higher ratios in early development of LGF (Supplementary Table S1). However, for the dynamic patterns of FA components, saturated (27.69% to 48.63%) and monounsaturated (19.80% to 37.72%) FAs showed the similar increasing trend with oil accumulation in developing LGF, but a down-trend for polyunsaturated (52.51% to 13.65%) FAs (Table 1). According to the temporal patterns of the contents and compositions for terpenoid and oil in developing LGF, 3 representative periods (50, 125 and 150 DAF) were characterized from 25–175 DAF for comparative deep transcriptomic analysis to better explore the molecular and metabolic regulatory mechanisms of the alternative carbon flux for terpenoid and oil accumulation in developing LGF.

### Transcriptome sequencing and unigene assembly

**Figure 2:**
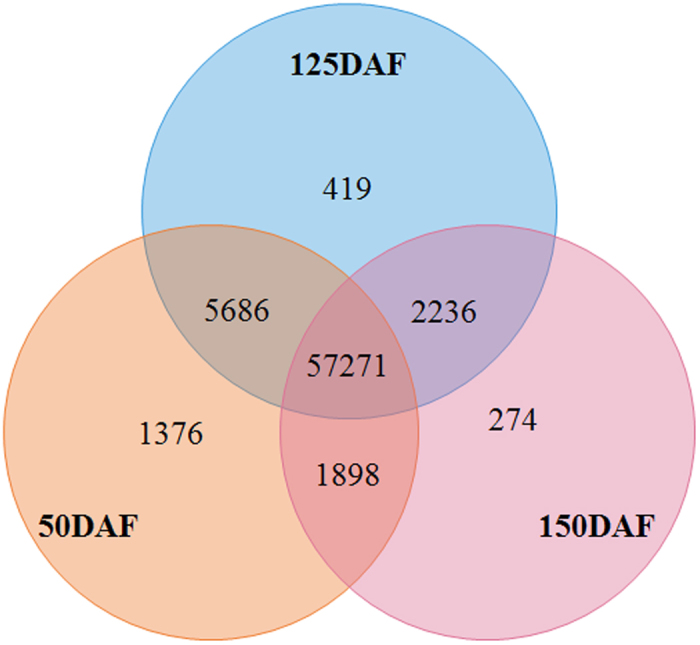
The unigene distribution at different development period.

To clarify a global overview of the gene expressing profiles in developing LGF, a total of three cDNA libraries were constructed based on the temporal accumulation of terpenoid and oil, and then were respectively sequenced by the Illumina 2000. After filtering the data by discarding low-quality reads and adaptor sequences, 28,021,984, 29,037,610 and 24,298,844 clean reads (average length = 92bp) were obtained from 50, 125 and 150 DAF (Table 2), respectively. The three transcriptome databases can be accessed in the Short Read Archive (SRA) under accession number SRR1952817, SRR1955000 and SRR1956757. Additionally, all the clean reads obtained from LGF of 3 developing stages were together assembled by the Trinity software, resulting in 69,160 unigenes with mean length of 665.79 bp (Supplementary Table S2). From the Venn diagram analysis, 57,272 unigenes were identified to be expressed in the whole developing stage, while 1376, 419 and 274 unigenes were specifically expressed in 50, 125 and 150 DAF, respectively (Fig. 2). The largest number of specific unigenes expressed at 50 DAF indicated more active processes involving in the differentiation and development of LGF in early stage, which was evidenced by the dynamic changes of LGF size (Supplementary Fig. S1). All the obtained unigenes could provide a substantial biology background for the exploration on the molecular regulatory mechanism of carbon source partitioning between terpenoid and oil accumulation in developing LGF.

### Transcript patterns for enzymes involved specially in TBP and OBP

All the *L. glauca* unigenes from three developmental stages were annotated by using the BLAST algorithm with an E-value <10^−5^ and protein identity >30% in public databases. Of the 69,160 unigenes, 14,942 (21.60%), 15,437 (22.32%), 15,529 (22.45%), 13,798 (19.95%), 14,689 (21.24%), 8248 (11.93%) unigenes showed significant similarities to known proteins in NR, SWISS-PROT, TREMBL, CDD, PFAM and COG database, respectively (Supplementary Table S3). Additionally, the species distribution in NR database showed that 6953 (46.53%) unigenes were high similar to the sequences from *Vitis vinifera*, followed by *Populus trichocarpa* (11.67%), *R. communis* (9.56%) and *Glycine max* (5.17%) (Supplementary Fig. 2). To further identify the interactions of all the annotated unigenes, we carried out GO functional enrichment and KEGG pathway analysis. The resulting 20,043 (28.98%) unigenes were assigned to the three main GO categories and 66 subcategories, among which ‘Cellular process’, ‘cell part’ and ‘binding’ were dominant subcategories in ‘biological process’, ‘cellular component’ and ‘molecular function categories’, respectively (Supplementary Fig. S3). Also, a total of 5,820 (8.42%) unigenes were assigned to 298 KEGG pathways and 754 kinds of enzymes (Supplementary Table S3). Notably, a total of 174, 71, 81 and 155 unigenes were implicated in glycolysis, PPP, EBP and OBP, respectively, indicating an intricate regulatory mechanism of carbon flux for oil and terpenoid biosynthesis in developing LGF.

**Figure 3:**
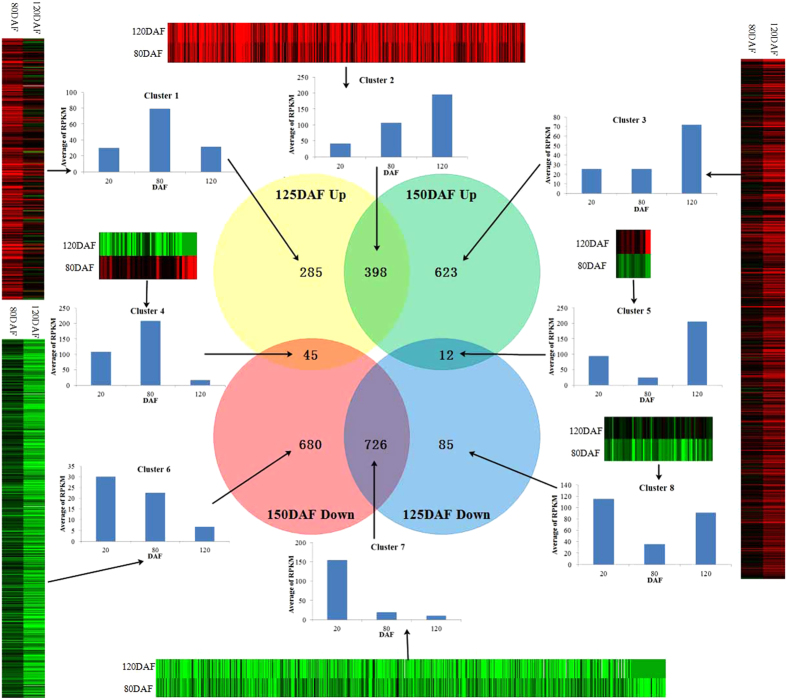
The number and distribution of up-regulated or down-regulated unigenes in developing LGF. The detailed sequences were showed in Tables S4.

To fully explore the differential unigene expressions in developing LGF, the clean reads from the three developing stages of LGF were mapped to our unigene database, and resulting 24.34 (86.84%), 25.71 (88.53%) and 21.29 (87.61%) million reads were perfect matched (Table 2). The normalization of gene expression data were performed by using multiple correction methods^44^ (Supplementary Fig. S4), and the differentially-expressed genes between the two LGF samples from different developmental stages were characterized using DESeq^45^ with *p*-value cut-off of 0.01 and using the BH method for multiple testing correction^46^, resulting in a total of 2854 unigenes identified with differentially expression in developing LGF (Supplementary Table S4). Notably, we characterized 370 and 1303 unigenes to be specific for 50:125 DAF and 50:150 DAF respectively (50 DAF as normalization), but 1096 unigenes shared in the both stages (Supplementary Table S4). According to their expression patterns, all the differentially-expressed unigenes were sorted into 8 clusters, including three up-regulated clusters (cluster 1, 2 and 3), three down-regulated clusters (cluster 6, 7 and 8) and two mixed clusters (cluster 4 and 5) (Fig. 3). Importantly, we characterized that the differentially expressed unigenes involving in glycolysis and PPP distributed in various clusters (cluster1, 2, 3, 7 and 8), whereas the TBP-specific unigenes were mainly focus on cluster 6 and 7, and OBP-specific unigenes on cluster 1 and 2. Therefore, the differential gene expression for the accumulation of terpenoid and oil may be specifically regulated in response to the circadian clock of developing LGF.

### Temporal allocation of carbon source for TBP and OBP

**Figure 4:**
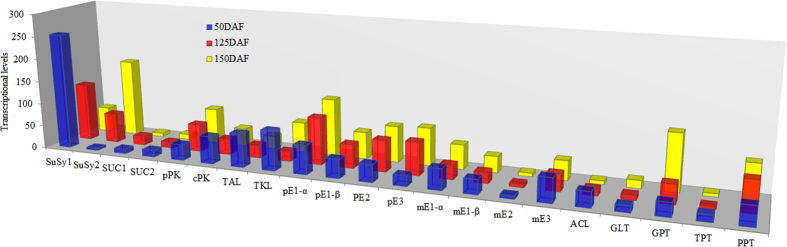
The transcriptional levels for the enzymes involved in the generation of G3P and acetyl-CoA.

**Figure 5:**
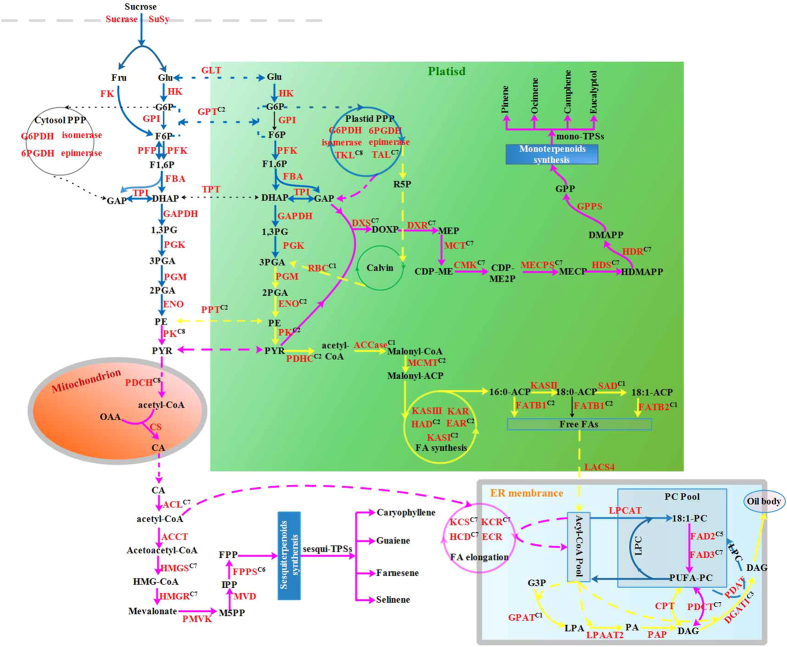
Compartmentalization of central carbon metabolism in developing LGF. Pink arrows show the main flux in early development. Yellow arrows show the main flux in middle-late development. Blue arrows show the main flux in the whole development. The superscripts show the results of differential-expressed cluster.

Sucrose is known as the most source of carbon required for terpenoid and oil biosynthesis in plants. To deeply understand the allocation of available carbon source for terpenoid and oil biosynthesis in developing LGF, the differential expression profiles of genes for key enzymes, providing the precursors for TBP and OBP, were concretely analyzed (Supplementary Tables S5, S6 and S7). We identified 2 unigenes for two SuSy isozymes with differential profiles (down in *SuSy1* and up in *SuSy2*) in developing LGF, but their expression levels were higher than those for two SUC isozymes (SUC1 and 2) with stable expression (Fig. 4), indicating that SuSy, as an important sucrose-cleaving enzyme, play a fundamental role in the supply of hexoses for the terpenoid and oil biosynthetic demand in developing LGF. Indeed, our qRT-PCR analysis attested the fact that *SuSy1* expression with a gradual decrease was observed in 7 developing stages of LGF, while *SuSy2* was gradually up-regulated (Supplementary Fig. S5). This striking difference of transcript level between two SuSy isozymes was entirely consistent with the different accumulative patterns of terpenoid and oil in developing LGF (Fig. 1), indicating that the expressions of *SuSy1* and *2* specifically responded to carbohydrate availability for terpenoid and oil biosynthesis in developing LGF. Additionally, we noticed the differential expression profiles of some crucial enzymes involved in carbon flux from the hexoses into TBP or OBP. Our data showed that the expressions of cytosolic *PK* and *ACL*, mitochondrial *PDHC*, plastidial *TKL* and *TAL* were all significantly up-regulated in early development of LGF, but a significant higher expression for plastidial *PK* and *PDHC* in middle-late development of LGF (Figs 4 and 5). These results revealed that a main hexose flux via cytosolic glycolysis or plastidial PPP is to provide acetyl-CoA or G3P respectively for terpenoid biosynthesis in early development of LGF, however a greater proportion of hexose to acetyl-CoA flux via plastidial glycolysis is required for oil biosynthesis in middle-late development of LGF. It was interesting to note that the genes encoding ribulose bisphosphate carboxylase (RBC), fixation of CO_2_ and ribulose 5-phosphate to 3-phospho-D-glycerate (3PGA)^47^, displayed significant higher expression at middle development of LGF (Supplementary Table S5). Combining with 3-fold higher expression of *ENO* in plastid than in cytosol (Supplementary Table S5), our findings indicated that Calvin pathway is crucial for the supplementation of carbon source for oil biosynthesis in middle development of LGF. Intriguingly, we also found that among the four glucolytic transporters (GLT, GPT, TPT and PPT), only both *PPT* and *GPT* showed a significant abundance of transcript in middle-late development of LGF (Fig. 4), as was in accordance with our qRT-PCR results that the expressions of *PPT* and *GPT* were up-regulated in 7 developing stages of LGF (Supplementary Fig. S5). Thus, a higher capacity of PPT and GPT provide glycolytic substrates (hexose phosphate, G6P and F6P) and intermediates (triose phosphate, PEP) from the cytosol to plastid for oil biosynthesis in developing LGF. Together, our data highlight that the partitioning of available carbon source (sucrose) is tightly regulated in response of LGF to different developing stages, leading to temporal allocation of sucrose flux to the important precursor (acetyl-CoA or G3P) required for TBP and OBP.

### A higher carbon flux into TBP in early developing LGF

To explore carbon flux from acetyl-CoA or G3P into TBP for terpenoid accumulation in developing LGF, the differential expression profiles of genes associated with terpenoid biosynthesis were analyzed, and our data revealed that most genes encoding the key enzymes (DXS, HMGCR, GPPS, FPPS, etc.) for terpenoid backbone biosynthesis (MVA and MEP pathway), and the genes for mono-TPSs and sesqui-TPSs, all displayed a higher transcriptional level in early development of LGF (Supplementary Table S6). This indicated that active biosynthesis of building blocks contribute to a rapid accumulation of terpenoid in early development of LGF, which is consistent with our findings on the temporal alternations of sucrose flux and terpenoid content at early phase (Fig. 1). These data reinforce and extend our previous conclusion that LGF are implicated actively in MVA and MEP pathway^43^. Surprisingly, a total of 6 and 4 unigenes showed high homology with the same enzyme of myrcene synthase (MS) and α-humulene synthase (α-HS) by which monoterpene (C10H16) and sesquiterpene (C15H24) hydrocarbons were synthesized, respectively. The phylogenetic analysis revealed that both *MS* and *α-HS* genes are clustered into 4 classes (Supplementary Fig. S6), showing an involvement of MS or α-HS in at least 4 different synthesis of monoterpene or sesquiterpene hydrocarbons, which can be confirmed by our detective results of the main four monoterpene hydrocarbons (α-pinene, β-pinene, β-cis-ocimene and camphene) and five sesquiterpene hydrocarbons (β-farnesene, cedrene-V6, α-selinene, β-selinene and farnesene) (Supplementary Table S1). Thus, the observed high sequence identity among MS and α-HS family members indicates the rapid evolution of a species-specific paralogous gene cluster in *L. glauca*. Together, our results of the higher expression levels for the enzymes in TBP reveal a higher carbon allocation ratio from sucrose to terpenoid accumulation in early developing LGF.

### A higher carbon flux into OBP in middle-late developing LGF

**Figure 6:**
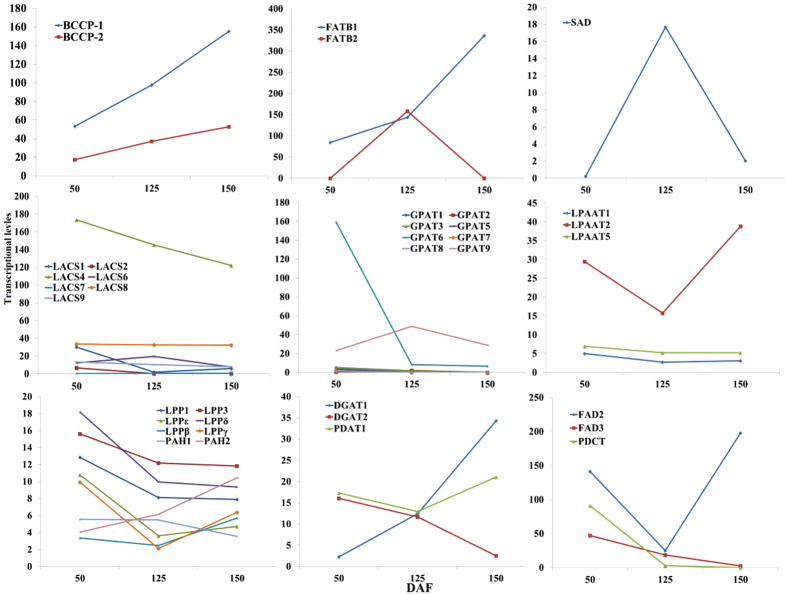
Temporal profile of transcriptional levels for enzymes involved in FA synthesis and TAG assembly.

In middle-late development of LGF, a greater proportion of hexose to acetyl-CoA flux is used for oil accumulation (Fig. 5). Indeed, the majority of our annotated genes associated with *de novo* FA synthesis were identified with a higher transcriptional level in middle-late development of LGF (Fig. 5 and Supplementary Table S7). It is, therefore, concluded that strongly increased FA synthesis, together with plastid carbon supply, is crucial for the higher oil accumulation in developing LGF. Notably, we characterized 2 genes for two BCCP isoforms (BCCP1 and BCCP2), but the abundance of *BCCP1* expression was 3-fold higher than *BCCP2* (Fig. 6), indicating a more important contribution of BCCP1subunit to control the carbon flux of acetyl-CoA to malonyl-CoA regulated by ACCase activity for *de novo* FA biosynthesis in developing LGF. The produced FA-ACP (16:0-, 18:0- and 18:1-ACP) must be hydrolyzed by FATB or FATA to release free FAs (saturated and monounsaturated) that are exported from the plastid required for TAG biosynthesis^26^. Unexpectedly, we only identified four unigenes encoding two FATB isozymes (FATB1 and FATB2) but no unigenes similar to FATA (Supplementary Table S7). Moreover, the analysis of transcriptional profiles revealed a gradual increased expression for *FATB1* in developing LGF, and a notably up-regulated expression for *FATB2* only at 125 DAF (comparable to expression pattern of *SAD6*), as was strongly supported by our qRT-PCR results of their expression alterations in 7 developing stages (Fig. 6 and Supplementary Fig. S5). Thus, we concluded that FATB1 and FATB2 are implicated respectively in regulation the flux of saturated (C16:0 and C18:0) and monounsaturated (C18:1) FAs from plastid to cytosol for TAG biosynthesis in developing LGF, which was also strengthened by our observation on palmitic (C16:0) and oleic acid (C18:1) as the major FA compositions (Table 1).

Considering the importance of the FA flux from cytosol to ER for FA elongation or esterification in developing LGF, we analyzed the differential expression profiles of the annotated 7 genes of LACS isozymes, and identified 5–10 fold higher expression level for *LACS4* than for the other *LACS* genes (Fig. 6), showing a preferred regulation for the FA flux to ER for oil accumulation by LACS4 in developing LGF. As for the biosynthesis of long chain FAs (more than 18 carbon), a series of FA elongases displayed higher transcriptional levels in early development of LGF (Supplementary Table S7), as were consistent with the high ratio of long-chain FA detected in early development of LGF (Table 1). The assembly of TAG from G3P and acyl-CoAs involves four enzymatic steps sequentially by GPAT, LPAT, PAP, and DGAT (or PDAT)^29^,^30^,^31^,^32^,^33^,^34^,^35^. As expected, we characterized 8 GPAT isoforms, but only *GPAT9* displayed a significant transcript in middle-late development of LGF (Fig. 6), indicating GPAT9 as the key enzyme for the initiation of TAG assembly. For the second step of TAG assembly by LPAAT and PAP, indeed, the genes for 3 and 8 isoforms of LPAAT and PAP were respectively identified in developing LGF, where *LPAT2* expression was 3–7 folds higher than *LPAAT1* and *LPAAT5*, whereas all of *PAP* showed no significant expression alteration (Fig. 6). These results indicate that LPAAT2 is as one major enzyme responsible for generation of PA for oil accumulation, and a coordinated expression of multiple PAP isozymes are contributing to DAG synthesis. As for the eventual TAG assembly, we characterized the genes high homology with *DGAT1* (up-regulated)*, DGAT2* (down-regulated) and *PDAT1* (stable expression) in developing LGF, which was confirmed by their corresponding changes detected in 7 developing stages by qRT-PCR (Supplementary Fig. S5). Our data revealed an involvement of the overlapping function (DGAT1 and PDAT1) in TAG production for oil accumulation of developing LGF, which was comparable to our previous results in developing seed kernels of Siberian apricot^43^. In addition, we characterized *PDCT* gene with up-regulated only in early development of LGF, and *CPT* with stable expression in developing LGF (Fig. 6). Integrated with the finding that the significant up-regulation of *FAD2/3* was parallel to the high percentage of polyunsaturated FAs in early development of LGF (Fig. 6 and Table 1), we conclude that a high expression of *PDCT* conduce to actively interconvert acyl residues between DAG and PC for further desaturation by FAD2/3, resulting in the accumulation of polyunsaturated FAs by PC-derived DAG synthesis in early development of LGF. In summary, our investigations could provide insight into which isoforms of these large gene families are responsible specifically for oil biosynthesis in developing LGF, and their high transcript levels evidence a higher carbon source partitioning from sucrose into OBP in middle-late developing LGF.

### Specifically transcriptional regulation for TBP and OBP

The TFs related to oil accumulation has been extensively studied, but little is known about TFs involving in regulation of terpenoid accumulation. By the differential expression analysis, we characterized 34 unigenes encoding 31 TFs belonging to 9 families, and the 10, 13 and 8 TFs with high expression were sorted into cluster-1, -2 and -3 in early, middle-late and whole development of LGF, respectively (Supplementary Table S8). Based on accumulative patterns of terpenoid and oil in developing LGF (Fig. 1), we suspected that the TFs in cluster-1 or -2 respectively regulated the terpenoid or oil biosynthesis in developing LGF, and the TFs of cluster-3 shared in both terpenoid and oil biosynthesis. In cluster-1, except for MYC2, the remaining 10 TFs (TT2, MYB1R1, CP19, NFYA9, NFYA7, MADS6, and bHLH93, 96 and 120) were not previously reported, and therefore they may be novel or specific for terpenoid biosynthesis of developing LGF. In cluster-2, we identified ABI3 and LEC1 as known TFs for oil biosynthesis, but the other 11 TFs (MYB90, NFYC2, bHLH82 and 148, WRKY23 and 65, and ERF5, 18, 26, 76, and 114) were novel or specific for developing LGF. It was noteworthy that the known WRI1 of cluster-3, displaying high expression levels in developing LGF, might play a pivotal role in expression regulation for some sugar-responsive genes with the consequence of alternative carbon partitioning from sucrose to terpenoid and oil in developing LFG. Additionally, the other novel 7 TFs in cluster-3 (GLK1, APL, MYB44, TCP2, RAP2-1 and 2–4, and ERF82) displayed the co-regulation for terpenoid and oil accumulation of developing LFG. Together, the above TFs with various expression patterns revealed the complex dynamic regulation mechanisms of carbon partitioning for terpenoid and oil accumulation in developing LGF.

## Discussion

Despite previous phytochemical studies of *L. glauca* has reported on the abundance of terpenoid and oil in fruits^1^,^2^,^8^, little is known about the molecular regulatory mechanisms of terpenoid and oil accumulation in developing LGF. Here, we firstly determinated the contents and compositions of terpenoid and oil in 7 developing stages of LGF (25–175 DAF), and characterized the accumulation of terpenoid and oil mainly at the early and middle-late development of LGF, respectively. To explore the regulatory mechanisms for the differential accumulation patterns between terpenoid and oil in developing LGF, we selected 3 crucial periods (50, 125 and 150 DAF) for comparative deep transcriptomic analysis for the first time, and identified some key genes implicated specifically in terpenoid and oil accumulation of developing LGF. It is well known that the carbon flux from sucrose to the precursors (G3P and acetyl-CoA) in plants for terpenoid or oil biosynthesis is regulated by some key metabolic enzymes, such as SuSy, HK, PFK, PK, PDHC, ACL, G6PDH, TKL and TAL^10^,^11^,^12^,^16^,^17^. By the combining analysis of differential expression profiling and qRT-PCR, we characterized that SuSy as the major enzyme initial the carbon metabolic flow for terpenoid and oil biosynthesis. Impressively, the cytosolic *PK* and *ACL*, mitochondrial *PDHC*, plastidial *TKL* and *TAL*, providing the G3P and acetyl-CoA for terpenoid biosynthesis^10^,^17^, all displayed up-regulated expression in early development of LGF, while the up-regulated expression was showed only for plastidial *PK* and *PDHC* in middle-late development, providing the acetyl-CoA for oil biosynthesis^16^. Therefore, combining with the temporal accumulation patterns of terpenoid and oil in 7 developing stages (Fig. 1), these data prompt us to conclude that LGF may allocate more carbon sources from sucrose into TBP in early development, whereas a higher assimilated carbon flux into OBP in middle-late development. Also consistent with this conclusion, all the genes involved in TBP or OBP showed up-regulated expressions in early or middle-late development of LGF, respectively (Fig. 5). Simultaneously, we characterized the involvement of 8 TPSs (4 MSs and 4 α-HSs) specificially in terpenoid biosynthesis, and the more contribution of BCCP2, FATB, LACS4, GPAT9, LPAAT2, DGAT1 and PDAT1 for oil biosynthesis in developing LGF. In addition to the above metabolic enzymes, 31 TFs with the significantly differential expressions were also identified to be highly related to developing LGF, including the known MYC2 for terpenoid biosynthesis^48^, ABI3 and LEC1 for oil biosynthesis^49^,^50^, and WRI1 involved in the activation of a subset of sugar-responsive genes for both terpenoid or oil accumulation^51^. In conclusion, our transcriptome sequencing and dynamic analysis revealed the complex regulatory mechanisms of carbon source partitioning between terpenoid and oil accumulation in developing LGF.

## Methods

### Collection of plant materials

*L. glauca* is widely distributed in China, so it has not been listed as an endangered or protected species and does not require approval. In this study, the different developmental stages of LGF were collected from Jigong Mountain (E114°06′, N32°125′) Natural Reserve of Henan Province. Flowers with the same anthesis were marked, and then seeds were harvested at 25 DAF (immature stage), 50 DAF, 75 DAF, 100 DAF, 120 DAF, 150 DAF and 175 DAF (fully matured stage) respectively. The samples were immediately frozen in liquid nitrogen and stored at −80 °C until use.

### Extraction of terpenoid and oil from developing LGK

About 50 g of the fresh LGF from each sample (approximate 15 samples per accession) were powdered ad subjected to hygro-distillationby using a modified Clevenger-type apparatus for five hours. The essential oil were collected, measured and dried over with anhydrous Na_2_oil_4_ and then stored in −20 °C until use. Oil was extracted with petroleum ether using a Soxhlet apparatus at 45–50 °C for 6–8 h until the extraction was completed. The oil content was determined as the difference in weight of the dried fruit sample before and after the extraction. The determination was performed in triplicate.

### GC-MS analysis

Terpenoid sample analyses were performed on an Agilent 7890Agas chromatograph, equipped with an Agilent 5975C quadrupole mass spectrometer and a HP-INNOWAX capillary column (60 m × 0.25 mm id, 0.25 μm film thickness J&W Scientific, Folsom, CA). Thetemperature program was as follows: from 50 °C (1 min hold) to 220 at 3 °C/min and then held at 220 °C for 5 min. The carrier gas was Helium was at 1 mL/min. The GC inlet was set in a splitting mode with split ratio 1:20 and at 230 °C and 1.0 μL of diluted samples (1/10, v/v, in hexane) were injected. Thequadrupole MS operating parameters: interface temperature 270 °C; electron impact ionization at 70 eV with scan mass range of 29–450 m/z. As for FA compositions, oil were trans-esterified under standard conditions employing a 5.5:1 molar ratio of methanol to oil using 1 wt% potassium hydroxide as a catalyst at 60–65 °C for 1 h. This was followed by a conventional work-up consisting of separation of phases, washing the resulting methyl esters with water until the water was neutral and drying with magnesium sulfate. The hexane (1 μL) extract was injected into a highly polar HP Innowax capillary column with a length of 30 m (inner diameter 0.32 m, film thickness 0.5 mm, split 1:20). An Agilent 6890 (CA, USA) equipped with a flame ionization detector was used. The injector and detector temperatures were 250 and 280 °C, respectively. The oven temperature was programmed from 190 °C holding at 3 min to 240 °C at the rate of 15 °C/min for 17 min. The carrier gas was high-purity hydrogen. The peaks were identified by comparing their retention time with that of the known standards, carried out under similar separation conditions. Peak integration was performed by applying HP3398A software. The above experiments were performed in triplicate.

### Total RNA extraction and transcriptome sequencing

Based on the detective results of LGF oil contents and FA compositions, the experimental LGF from the 3 crucial periods (50, 120 and 150 DAF) were selected as materials for transcriptome sequencing. The equal weight of 5–10 biological samples from every developmental stage were mixed, and then total RNA was extracted from the mixture using RNeasy Plant Mini Kits (Qiagen, Inc., Valencia, CA, USA) according to the manufacturer’s protocol. Extracted RNA was qualified and quantified using a Nanodrop ND-1000 Spectrophotometer (Nanodrop Technologies, Wilmington, DE, USA) and all the samples showed a 260/280 nm ratio from 1.9 to 2.1. The equivalent RNA samples for transcriptome analysis were prepared using Illumina’s kit following manufacturer’s protocol (Illumina, San Diego, CA, USA). The cDNA library was sequenced on the Illumina sequencing platform (HiSeqTM 2000), and two technical repetitions were performed. After removal of the adapter sequences, the low quality sequences (reads with ambiguous bases ‘N’) and reads with more than 10% Q < 20 bases, all the clean reads were together assembled with the Trinity program, and the Trinities were clustered using TGICL tools into unigenes^52^.

### Sequence annotation

To understand their functions, the LGF unigenes were annotated using BLASTX alignment with an E-value cut-off of 10^−5^ against the following protein databases: NR, SWISS-PROT, TREMBL, CDD, PFAM and COG database. Functional annotation by GO terms (http://www.geneontology.org) was analyzed by Blast2Go software. The KEGG pathways annotation was performed using BLAST all against Kyoto Encyclopedia of Genes and Genomes database.

### Differential expression analysis of unigenes

According to the previous identification of appropriate reference genes for normalizing transcript expression in developing fruits of *Litsea cubeba*, belonging to the same family (Lauraceae) with our experimental materials^53^, 9 reference genes with the pairwise variation of 0.08 were used for normalizing our transcriptome data (Supplementary Table S9). The normalization of gene expression data were performed by using multiple correction methods^44^, and the distribution of RPKMs before and after correction were shown in Supplementary Fig. S4. Subsequently, the data from the reference gene-based correction on the RPKM were used for differential expression analysis by using DESeq^45^ with p-value cut-off of 0.01 and using the BH method for multiple testing correction^46^.

### qRT-PCR assay

Total RNA was extracted as the description for the cDNA library preparation and was reverse transcribed using the Reverse transcription System (Promega). The amplification primers were designed using PrimerQuest (http://www.idtdna.com/PrimerQuest/Home/Index) software with melting temperatures at 62 °C, and the absence of secondary structures was verified by the UNAFold program (http://eu.idtdna.com/UNAFold) according to D’haene *et al*.^54^. Also, large subunit ribosomal protein L32e and ubiquitin-conjugating enzyme genes were used as internal controls. The primers list in Supplementary Table S10. The qRT-PCR was performed using the SYBR Premix Ex Taq Kit (TaKaRa) according to the manufacturer’s protocol. Negative controls consisting of nuclease-free water instead of template, and reverse transcriptase controls prepared by substituting reverse transcriptase for nuclease-free water in the cDNA synthesis step were included in all analyses for each primer pair. Three technical repetitions were performed for qRT-PCR.

## Additional Information

**How to cite this article**: Niu, J. *et al*. Integrated transcriptome sequencing and dynamic analysis reveal carbon source partitioning between terpenoid and oil accumulation in developing *Lindera glauca* fruits. *Sci. Rep*. **5**, 15017; doi: 10.1038/srep15017 (2015).

## Supplementary Material

Supplementary Information

Supplementary Dataset 1

Supplementary Dataset 2

Supplementary Dataset 3

Supplementary Dataset 4

Supplementary Dataset 5

Supplementary Dataset 6

Supplementary Dataset 7

## Figures and Tables

**Table 1:** The percentage of FAs in developing LGF.

DAF	C10:0(%)	C12:0(%)	C16:0(%)	C16:1(%)	C18:0(%)	C18:1(%)	C18:2(%)	C18:3(%)	C20:4(%)	Saturated FAs(%)	Monounsaturated FAs(%)	polyunsaturated FAs(%)
25	—	—	21.22 ± 1.31	—	4.47 ± 0.24	21.80 ± 1.58	29.75 ± 2.88	7.27 ± 0.74	15.49 ± 1.35	25.69	21.8	52.51
50	2.77 ± 0.20	1.59 ± 0.18	22.61 ± 1.85	—	5.12 ± 0.41	21.19 ± 2.31	28.70 ± 4.06	6.44 ± 0.47	11.58 ± 1.55	32.09	21.19	46.72
75	12.49 ± 1.38	5.22 ± 0.56	22.33 ± 1.99	—	3.05 ± 0.16	30.76 ± 4.33	20.17 ± 2.06	0.77 ± 0.09	5.21 ± 0.46	43.09	30.76	26.15
100	16.28 ± 1.43	8.24 ± 1.18	22.06 ± 2.38	1.08 ± 0.06	1.03 ± 0.14	37.14 ± 2.64	11.38 ± 1.40	0.74 ± 0.06	2.05 ± 0.14	47.61	38.22	14.17
125	10.35 ± 1.33	5.83 ± 0.48	30.22 ± 3.05	1.65 ± 0.08	0.95 ± 0.10	28.80 ± 5.47	11.44 ± 1.25	0.76 ± 0.08	—	47.35	42.45	10.2
150	11.08 ± 1.23	5.40 ± 0.36	31.15 ± 3.58	1.57 ± 0.12	0.88 ± 0.06	37.20 ± 3.26	12.07 ± 0.72	0.65 ± 0.09	—	48.51	38.77	12.72
175	11.83 ± 1.00	5.80 ± 0.48	30.13 ± 2.73	1.54 ± 0.09	0.87 ± 0.12	36.18 ± 2.76	12.92 ± 1.23	0.73 ± 0.09	—	48.63	37.72	13.65

**Table 2:** Reads data statistics.

DAF	Trim reads	Total length(bp)	Average length(bp)	Map reads	Map reads%
50	28,021,984	2,598,396,941	92.73	24,335,597	86.84%
125	29,037,610	2,681,376,343	92.34	25,707,321	88.53%
150	24,298,844	2,233,238,833	91.91	21,287,874	87.61%
