# Supplementary material for: Integrated transcriptome sequencing and dynamic analysis reveal carbon source partitioning between terpenoid and oil accumulation in developing Lindera glauca fruits
Source: Sci Rep. 2015 Oct 8;5:15017. doi: 10.1038/srep15017 (PMC4597268; doi:10.1038/srep15017)
Supplement: Supplementary Information [file srep15017-s1.doc]

**Integrated transcriptome sequencing and dynamic analysis reveal** **carbon source partitioning between terpenoid and oil accumulation in developing *Lindera glauca* fruits**

**Jun Niu^1^,** **Yinlei Chen^1^, Jiyong An^1^, Xinyu Hou^1^, Jian Cai^1^, Jia Wang^1^,** **Zhixiang Zhang^1^,** **Shanzhi Lin^1*^**

^1^College of Biological Sciences and Biotechnology, College of Nature Conservation, National Engineering Laboratory for Tree Breeding, Key Laboratory of Genetics and Breeding in Forest Trees and Ornamental Plants, Ministry of Education, Beijing Forestry University, Beijing 10083, China

^*^Corresponding author: Tel/Fax +86-10-62336114; szlin@bjfu.edu.cn


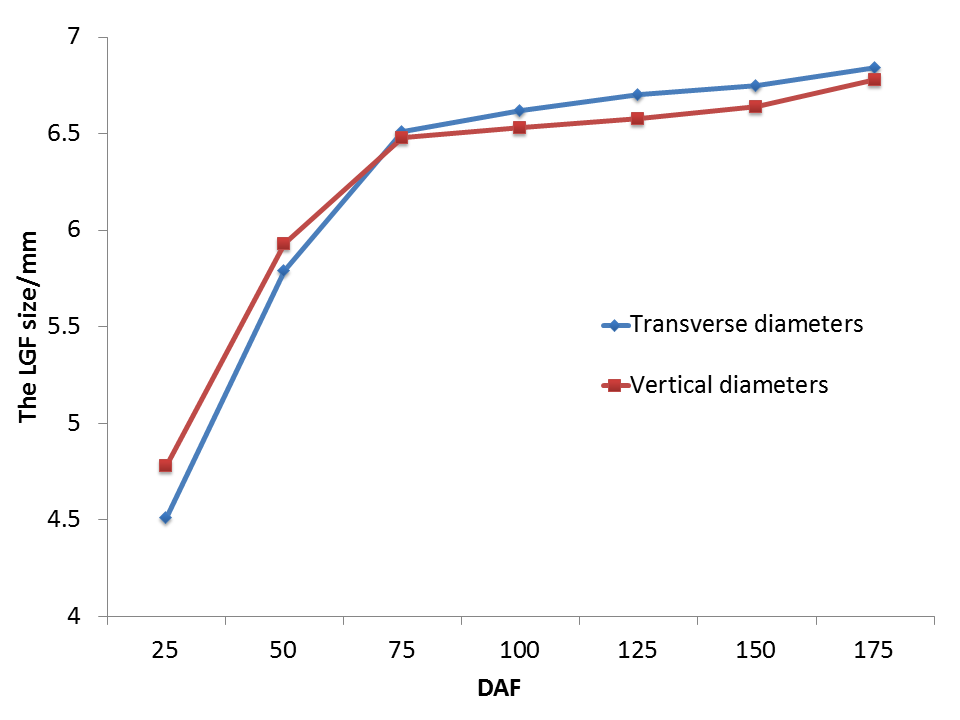


**Figure.S1** The fruit size in different [developmental](javascript:void(0);) [stage.](javascript:void(0);)


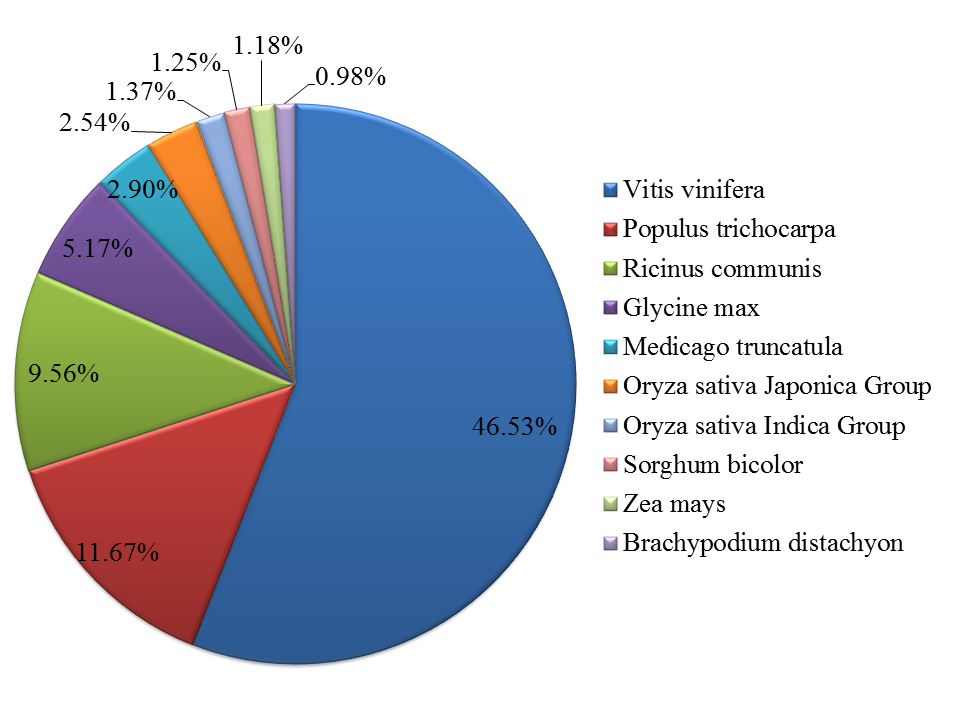


**Figure.S2** Species distribution of the top BLAST hits for reads in Nr database.


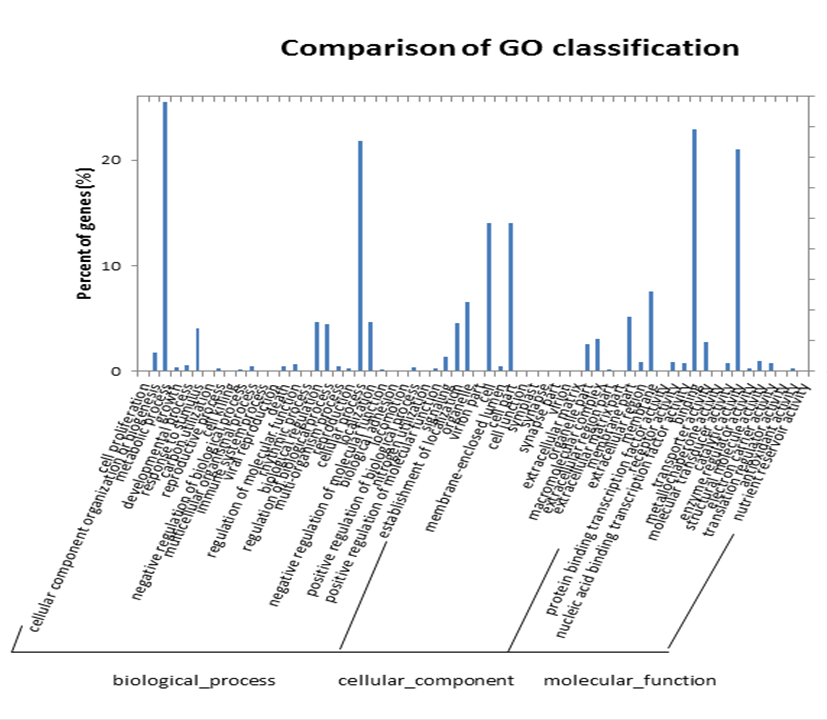


**Figure.S3** Histogram presentation of Gene Ontology classification.


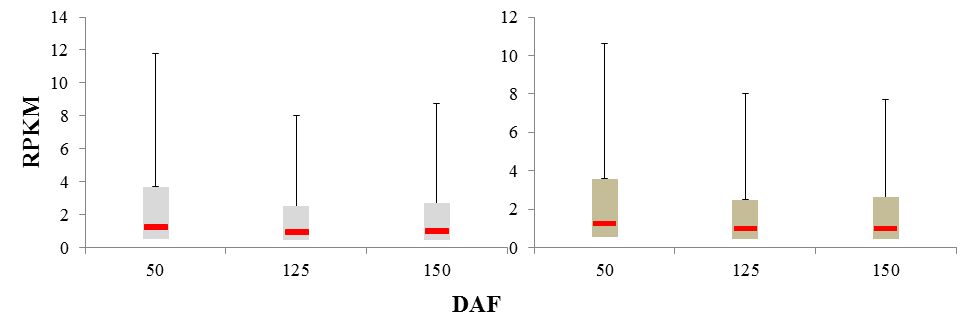


**Figure.S4** Transcript RPKM distribution before (left) and after correction (right).


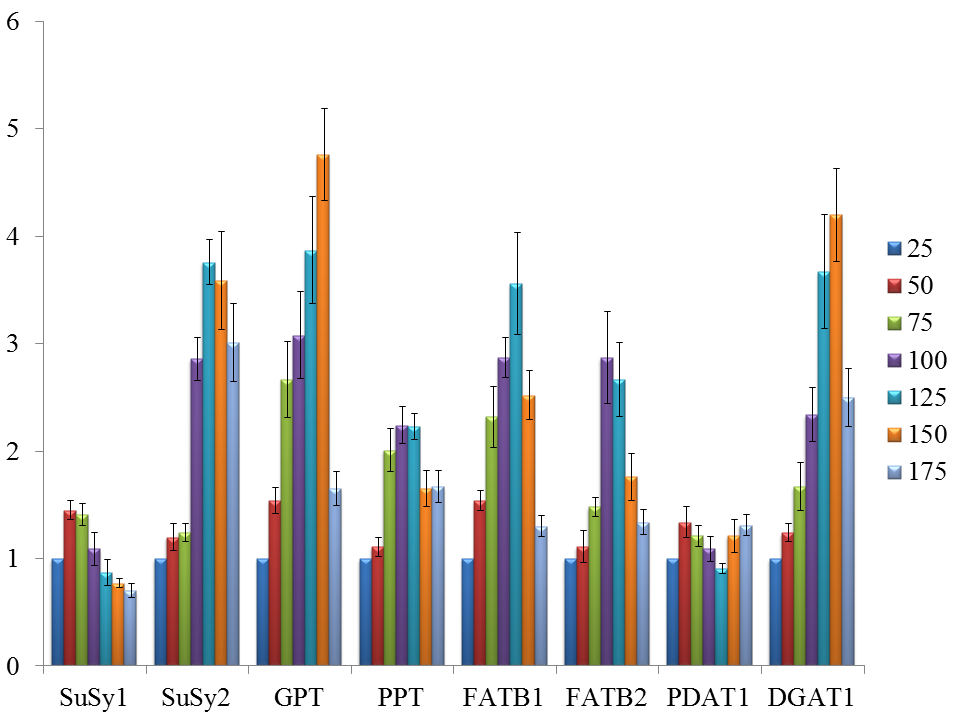


**Figure.S5** Gene expression data obtained by qRT-PCR analysis.


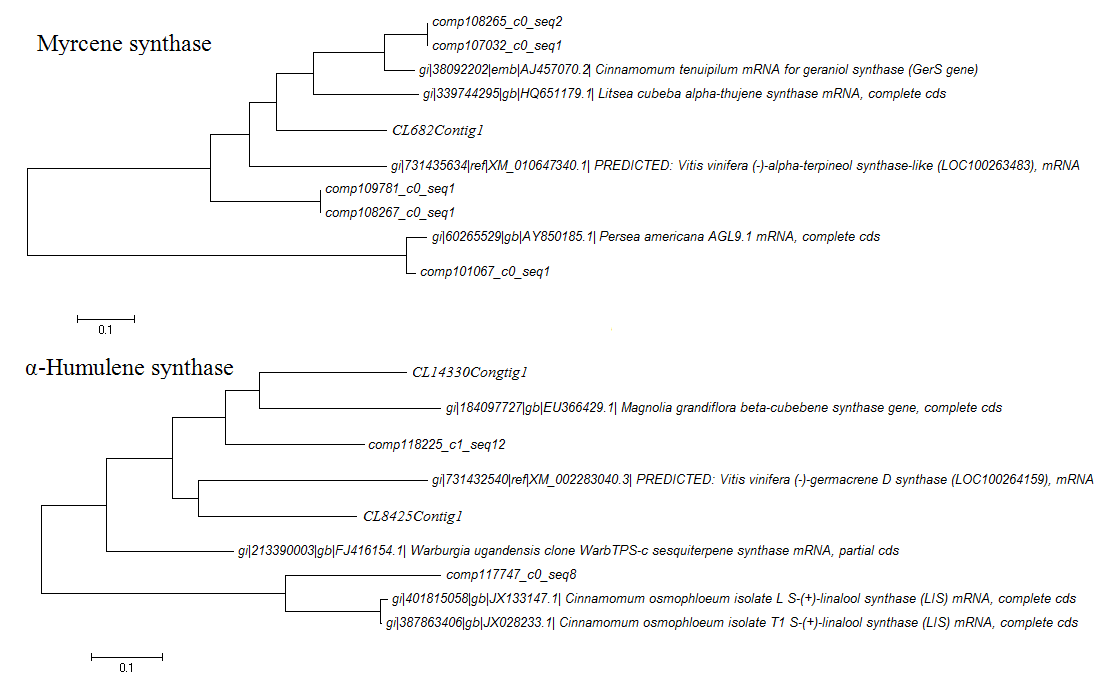


**Figure.S6** The phylogenetic analysis of MS and α-HS genes.

**Table S2**

**The length distribution and quality evaluation of unigenes**

|  | **All (>=200bp)** | **>=500 bp** | **>=1000 bp** | **Total Length** | **Max Length** | **Min Length** | **Average Length** |
| --- | --- | --- | --- | --- | --- | --- | --- |
| **Transcript** | 256,654 | 107,399 | 57,780 | 185,003,079 | 9361 | 201 | 720.83 |
| **Unigene** | 69,160 | 26,099 | 12,572 | 46,046,507 | 9361 | 201 | 665.79 |

**Table S9**

**The reference genes used for normalizing our transcriptome data**

| name | GenBank accession number | *Arabidopsis* homolog locus |
| --- | --- | --- |
| Elongation factor 1-alpha | KF706375 | AT5G60390 |
| Eukaryotic initiation factor 4-alpha | KF706376 | AT1G72730 |
| Actin-7 | KF706373 | AT5G09810 |
| Actin-11 | KF706374 | AT3G12110 |
| F-box protein | KF706377 | AT3G53000 |
| Translationally-controlled tumor protein | KF706380 | AT3G16640 |
| Tubulin alpha | KF706381 | AT4G14960 |
| Tubulin beta | KF706382 | AT5G12250 |
| Ubiquitin-conjugating enzyme | KF706383 | AT5G53300 |

**Table S10**

The information of primers for qRT-PCR.

| Unigene | name | abbreviation | Forward Primer | Reverse Primer | Ampliconsize (bp) |
| --- | --- | --- | --- | --- | --- |
| comp108110_c1_seq1 | sucrose synthase1 | SuSy1 | TCTCATCTTGCTCCTCCCTATC | CTGGATTGGTAGAGTGGTTTGG | 114 |
| comp109010_c1_seq2 | sucrose synthase2 | SuSy2 | TGCAGATTGAAGTCGGGATAAG | CCTTCGAGCCCACAAATACA | 79 |
| comp94137_c0_seq1 | phosphoenolpyruvate transporter | PPT | ATTGTCCAGGGATTCCTCTTTC | GGAGTGCGATGGCTTCTAAT | 89 |
| comp104125_c0_seq1 | glucose-6-phosphate transporter | GPT | CGGATCTTTGGAAGGATTGAGA | GTCAGGGATCTGGACGTTAATC | 103 |
| comp101094_c0_seq2 | Fatty acyl-ACP thioesterase B1 | FATB1 | TCAAGGGTCATGCAGGAAAG | CTGGAGCGATCTGGATGTTAAT | 117 |
| comp107319_c0_seq1 | fatty acyl-ACP thioesterase B2 | FATB2 | TTGCAGGAGGCTACACTTAATC | CAACCCACATCAGATCTCTCTTAC | 100 |
| comp104268_c0_seq1 | phospholipid: diacylglycerol acyltransferase | PDAT1 | CAGCTGATAATGGGACAGTGAG | TGCCTCTGCCACATCTTTAC | 92 |
| comp110824_c0_seq2 | acyl-CoA:diacylglycerol acyltransferase | DGAT1 | GCACCATCTGCACTCACATA | GGTATCCTATGCCCACACAAA | 96 |
| comp112461_c0_seq1 | ubiquitin-conjugating enzyme | UBC | CTGGGATACCATCCAGAACATC | CTCAAGTGTCCTTCCAGCATAG | 97 |
| comp90151_c0_seq1 | large subunit ribosomal protein L32e | RPL32 | CCGCCACCTCTCTCTTTATTT | GCGCTTCTTGACAATCTTCTTG | 102 |
